# Short‐term semaglutide treatment improves FGF21 responsiveness in primary hepatocytes isolated from high fat diet challenged mice

**DOI:** 10.14814/phy2.15620

**Published:** 2023-03-10

**Authors:** Jia Nuo Feng, Weijuan Shao, Tianru Jin

**Affiliations:** ^1^ Department of Physiology, Temerty Faculty of Medicine University of Toronto Toronto Canada; ^2^ Division of Advanced Diagnostics, Toronto General Hospital Research Institute University Health Network Toronto Canada

**Keywords:** FGF21, FGF21 resistance, FGFR1, KLB, semaglutide

## Abstract

Metabolic functions of GLP‐1 and its analogues have been extensively investigated. In addition to acting as an incretin and reducing body weight, we and others have suggested the existence of GLP‐1/fibroblast growth factor 21 (FGF21) axis in which liver mediates certain functions of GLP‐1 receptor agonists. In a more recent study, we found with surprise that four‐week treatment with liraglutide but not semaglutide stimulated hepatic FGF21 expression in HFD‐challenged mice. We wondered whether semaglutide can also improve FGF21 sensitivity or responsiveness and hence triggers the feedback loop in attenuating its stimulation on hepatic FGF21 expression after a long‐term treatment. Here, we assessed effect of daily semaglutide treatment in HFD‐fed mice for 7 days. HFD challenge attenuated effect of FGF21 treatment on its downstream events in mouse primary hepatocytes, which can be restored by 7‐day semaglutide treatment. In mouse liver, 7‐day semaglutide treatment stimulated FGF21 as well as genes that encode its receptor (FGFR1) and the obligatory co‐receptor (KLB), and a battery of genes that are involved in lipid homeostasis. In epididymal fat tissue, expressions of a battery genes including *Klb* affected by HFD challenge were reversed by 7‐day semaglutide treatment. We suggest that semaglutide treatment improves FGF21 sensitivity which is attenuated by HFD challenge.

## Graphical abstract

we revealed in this study that short‐term semaglutide treatment stimulates liver FGF21 expression and the sensitivity of FGF21.

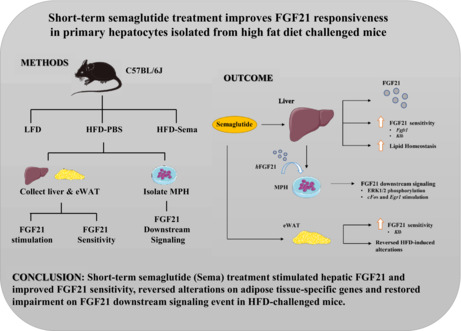

**New and Noteworthy**

The novel GLP‐1/FGF21 axis has been suggested to play an important role in mediating beneficial effects of GLP‐1‐based drugs in obese subjects. We show here that 7‐day semaglutide treatment stimulated hepatic FGF21 expression and improved FGF21 sensitivity. Our findings have deepened our mechanistic understanding on functions of GLP‐1‐based drugs and the pathophysiological importance of the GLP‐1/FGF21 axis.

## 1 INTRODUCTION

Incretins are defined as gut‐produced hormones that function to augment insulin secretion from pancreatic β cells in a glucose‐concentration‐dependent manner (Tian & Jin, 2016). Glucagon‐like peptide‐1 (GLP‐1), produced by gut endocrine L cells, was recognized as the 2nd incretin in the middle of the 1980s (Baggio & Drucker, 2007; Holst, 2007; Kieffer & Francis Habener, 1999; Muller et al., 2019; Pederson & McIntosh, 2016; Petersen & Shulman, 2018; Tian & Jin, 2016). Following the recognition that GLP‐1 serves as an incretin, various incretin or GLP‐1‐based drugs including GLP‐1 receptor (GLP‐1R) agonists (GLP‐1RAs) and dipeptidyl peptidase‐4 inhibitors (DPP‐4i) have been developed for treating type 2 diabetes (T2D). Among them, liraglutide (Victoza®) and semaglutide (Ozempic®) are now FDA‐approved therapeutic agents for both T2D and chronic weight management (Deacon, 2020; Garber, 2011; Hinnen, 2017). Pieces of puzzles are still missing to explain how beneficial effects of them can be seen in subjects with severe insulin resistance if GLP‐1RAs purely act as incretin. Efforts have been made in numerous studies, showing the existence of extra‐pancreatic functions of GLP‐1 and GLP‐1RAs (Chiang et al., 2013, 2014; Chiang & Jin, 2014; Ip et al., 2012, 2013, 2015; Jin, 2016; Jin & Weng, 2016; Shao et al., 2015, 2020; Shao, Wang, Chiang, et al., 2013; Shao, Wang, Ip, et al., 2013; Zhou et al., 2020).

GLP‐1R is expressed in tissues including pancreas, lung, heart, gastric intestinal (GI) tract, brain, and kidney, which allows GLP‐1 or GLP‐1RAs to exert their metabolic and other effects directly (Pang et al., 2022; Viby et al., 2013; Zhou et al., 2020). In white adipose tissues (WAT), the effect of GLP‐1 or GLP‐1RAs could be mediated by a small portion of GLP‐1R^+^ cells of stromal vascular fraction (SVF), or certain lymphocytes or endothelial cells that do express GLP‐1R (Gu et al., 2022; McLean et al., 2021). Although hepatic functions of GLP‐1 and GLP‐1Rs on both glucose and lipid homeostasis have been broadly recognized, it is unlikely that GLP‐1R is expressed in hepatocyte (Jin & Weng, 2016; Liu et al., 2021; Panjwani et al., 2013). Hence, GLP‐1 and GLP‐1RAs may exert their hepatic functions either indirectly or via a small portion of GLP‐1R^+^ cells in the liver that are hematopoietic or endothelial origins (Jin & Weng, 2016; Liu et al., 2021; McLean et al., 2021).

Although the gene that encodes fibroblast growth factor 21 (FGF21) can be detected in mouse liver, adipose tissues, pancreatic islets, and elsewhere, circulating FGF21 is considered liver driven (Badakhshi & Jin, 2021). It is defined as metabolic hormone due to the lack of the conventional heparin‐binding domain. Since the discovery of FGF21, extensive investigations have been conducted on determining its role in metabolic homeostasis. FGF21 mediates its function through the binding of the heterodimeric receptor complex comprising mainly FGF receptor 1 or 3 (FGFR1 or FGFR3) and the obligatory co‐receptor, β‐klotho (KLB; Badakhshi & Jin, 2021; Geng et al., 2020; Shao et al., 2022). Metabolic beneficial effects of FGF21 have been established in both pre‐clinical investigations and in various clinical trials (Badakhshi & Jin, 2021; Shao & Jin, 2022). FGF21 knockout (KO) mice fed with ketogenic diet demonstrated impaired glucose tolerance with fatty liver and altered hepatic gene expression. Replenish FGF21 KO mice with human recombinant FGF21 (hFGF21) can attenuate dietary challenge‐induced metabolic impairment (Badman et al., 2009; Li et al., 2018).

Studies have shown that in various rodent models, GLP‐1RAs can positively regulate hepatic FGF21 production (Lee et al., 2014; Liu et al., 2019; Nonogaki et al., 2014; Yang et al., 2012). We have reproduced such observation and demonstrated that indeed GLP‐1R is not expressed in mouse liver, that in GLP‐1R KO mice, liraglutide virtually lost its metabolic beneficial effect and cannot stimulate liver FGF21 expression, and that in liver‐specific FGF21 KO mice, metabolic beneficial effects of liraglutide were severely attenuated (Liu et al., 2021). We hence suggest that this novel GLP‐1/FGF21 axis is patho‐physiologically important (Liu et al., 2021). In a more recent follow‐up study, we compared the effect of liraglutide and semaglutide on hepatic FGF21 expression in HFD‐challenged mice. Surprisingly, semaglutide, the long‐term effective GLP‐1RA showed no effect on stimulating hepatic FGF21 expression, although its metabolic homeostatic effects are highly appreciable (Liu et al., 2022). We hence wonder whether semaglutide can also improve FGF21 sensitivity or responsiveness and hence triggers a negative feedback loop in attenuating its stimulatory effect on hepatic FGF21 expression. Here, we assessed short‐term effect of semaglutide treatment in HFD‐challenged mice. Our observations indicate that daily semaglutide treatment for 7‐day increased hepatic FGF21 expression. The treatment also attenuated HFD challenge‐induced repression on *Klb* in both liver and white adipose tissue. More importantly, mouse primary hepatocyte (MPH) from HFD‐challenged mice showed attenuated response to hFGF21 treatment, while MPH from HFD‐fed mice with 7‐day semaglutide treatment showed improved response to hFGF21 treatment.

## 2 MATERIALS AND METHODS

### 2.1 Chemicals

Recombinant human FGF21 (hFGF21) was purchased from Cayman Chemical. Semaglutide was kindly provided by Novo Nordisk, as we have reported (Liu et al., 2022).

### 2.2 Animals and animal experiments

Six‐week‐old male C57BL/6J mice, purchased from the Jackson laboratory, were either fed with low fat diet (LFD) or HFD (F3282 with 60% fat calories, 5.49 kcal/g; BioServ. Flemington, New Jersey) for 13 weeks, followed by daily intraperitoneal (*i.p*.) semaglutide (600 μg/kg body weight) or control PBS injection for 1 week. By the end of dietary challenge and semaglutide treatment, mice were sacrificed with CO_2_ treatment followed by cervical dislocation. Plasma and tissues, including the liver, epididymal WAT (eWAT), inguinal WAT (iWAT), and brown adipose tissue (BAT), were collected for real‐time RT‐PCR and Western blotting. Mice were housed at constant temperature (22°C) under restricted light cycle with food and water *ad labitum*. The animal experiments were approved by the University Health Network Animal Care Committee Animal Resource Center (AUP# 2949.13).

### 2.3 Glucose tolerance test, plasma adiponectin, and leptin measurements

For *i.p*. glucose tolerance test (IPGTT), mice were fasted overnight prior to receiving *i.p*. glucose injection (2 g/kg body weight). Mouse plasma adiponectin level was measured utilizing the mouse adiponectin immunoassay kit (Antibody and Immunoassay Services, The university of Hong Kong). The measurement of plasma leptin was conducted utilizing Mouse Leptin DuoSet ELISA Kit (R&D, USA; Tian et al., 2020).

### 2.4 Mouse primary hepatocyte (MPH) isolation and hFGF21 treatment

MPH from indicated mice were isolated as we have reported previously (Liu et al., 2021). For real‐time RT‐PCR in assessing the effect of hFGF21 on gene expression, cells were treated with indicated dose of hFGF21 for 4 h. For Western blotting in determining effects of hFGF21 on AKT or ERK phosphorylation, cells were treated with indicated dose of hFGF21 for 1 h.

### 2.5 RNA extraction, quantitative reverse transcription PCR

Tri reagent (Sigma‐Aldrich) was used for RNA isolation. RNA extraction, reverse transcription, and real‐time polymerase chain reaction (PCR) was performed as previously described (Shao et al., 2020). Primer sequences utilized for PCR are listed in Table 1.

**TABLE 1:** Nucleotide sequences of primers utilized.

Gene name (mouse)	Forward sequence (5′–3′)	Reverse sequence (5′‐3′)	Size (bp)
*cFos*	TCCCCAAACTTCGACCATG	GCACTAGAGACGGACAGATC	189
*Egr1*	CAACCCTATGAGCACCTGAC	CCACTGACTAGGCTGAAAAGG	197
*Fgfr1*	GTGGAGAATGAGTATGGGAGC	GGATCTGGACATACGGCAAG	238
*Klb*	ACGAGGGCTGTTTTATGTGG	CAGGTGAGGATCGGTAAACTG	226
*Acox1*	CAGGAAGAGCAAGGAAGTGG	CCTTTCTGGCTGATCCCATA	189
*Pdk4*	ACATCGCCAGAATTAAACCTCAC	TTTCCCAAGACGACAGTGGC	191
*Ehhadh*	AGCTGTTTATGTACCTTCGGG	CTGCTTTGGGTCTGACTCTAC	236
*Ppargc1α*	TGGATGAAGACGGATTGCCC	GTGTGGTTTGCTGCATGGTT	220
*Fasn*	AGAAGTGCAGCAAGTGTCC	GGTCGGATGAGGGCAATCTG	258
*Srebf1*	TAGAGCATATCCCCCAGGTG	GGTACGGGCCACAAGAAGTA	245
*Chrebp*	CCCCCAGCTTTGGCCCCATG	TCGGTCCAGGAGCAGGTGGG	234
*Ctp1α*	AGATCAATCGGACCCTAGACAC	CAGCGAGTAGCGCATAGTCA	122
*Ucp1*	GGGCCCTTGTAAACAACAAA	GTCGGTCCTTCCTTGGTGTA	196
*AdipoQ*	AGAAGCCGCTTATGTGTATC	TGATACTGGTCGTAGGTGAA	255
*Lep*	CCTGTGGCTTTGGTCCTATC	TCATTGGCTATCTGCAGCAC	273
*Ero1α*	TTCTGGGCGAGGAAAAAGTA	TGACCCCATTTCTTTTCCAG	171
*Erp44*	TGTGCCTTCCTTTCTGCTTT	CGGACAAGAGGGACACATTT	173
*Actb*	TCATGAAGTGTGACGTTGACA	CCTAGAAGCATTTGCGGTG	285

### 2.6 Western blotting

Whole‐cell lysates from mouse liver, indicated adipose tissue, or MPH were prepared for Western blotting as previously described (Tian et al., 2019). Antibodies for western blotting were listed in Table 2. Membranes were visualized using Pierce ECL Western Blotting Substrate (Thermo Scientific). Image densitometries were analyzed using ImageJ 1.53 software.

**TABLE 2:** Antibodies utilized in the current study.

	Dilution	Company	Catalog #
pERK	1:2000	Cell Signaling Technology	9106S
Total ERK	1:1000	Santa Cruz	SC‐94
pAKT	1:1000	Cell Signaling Technology	SC‐293125
Total AKT	1:1000	Santa Cruz	9272S
FGF21	1:1000	Abcam	ab171941
GAPDH	1:1000	Cell Signaling Technology	2118

### 2.7 Statistical analysis

Results are expressed as mean ± SD. Differences between multiple groups were analyzed by one‐way ANOVA followed by Bonferroni post hoc tests or unpaired student's *t*‐test. A *p*‐value less than 0.05 is considered as significantly different.

## 3 RESULTS

### 3.1 Seven‐day semaglutide treatment reduces body weight and improves glucose tolerance in HFD‐challenged mice

**FIGURE 1:**
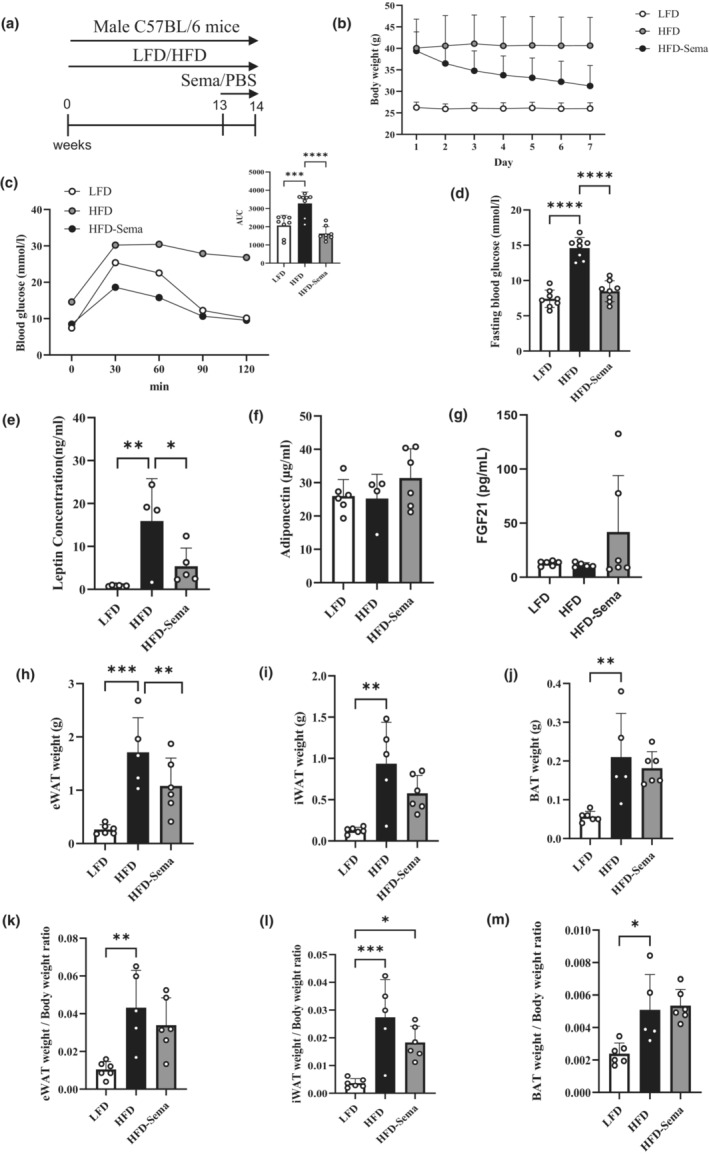
Short‐term semaglutide treatment improves glucose tolerance and reduces body weight in HFD‐challenged mice. (a) Diagram shows the animal experimental design. (b) Body weight changes during last 7 days in the three indicated groups. (c) Blood glucose level and area under the curve (AUC) during IPGTT. (d) Fasting (overnight) blood glucose levels at the end of the experiment for indicated groups. (e–g) Fasting plasma leptin (e), adiponectin (f), and FGF21 (g) levels. (h–m) Fat pad weights including epididymal (h, eWAT) and inguinal (i, iWAT) white adipose tissue and brown adipose tissue (j, BAT). (k) eWAT weight to body weight ratio. (l) iWAT weight to body weight ratio. (m) BAT weight to body weight ratio. Sema, semaglutide. Data are shown as the mean ± SD. *p < 0.05, **p < 0.01, ***p < 0.001, ****p < 0.0001.

As shown (Figure 1a), we aimed to test metabolic beneficial effects of short‐term (but high dose) semaglutide treatment in obese mice, generated by HFD challenge. Male C57BL/6J mice were fed with LFD or HFD for 13 weeks. HFD‐challenged mice were then randomly divided into two sub‐groups, receiving either daily semaglutide *i.p*. (600 μg/kg body weight) or PBS (as control) injection for 7 days. The treatment generated profound body weight lowering effect (Figure 1b and Supporting Figure S1A,B). Food intake was significantly decreased in mice received seven‐day semaglutide treatment (Supporting Figure S1C). Figure 1c shows that the treatment also improved glucose disposal, assessed by IPGTT. HFD‐induced elevation on fasting glucose level as well as hyperleptinemia were also reversed by 7‐day semaglutide treatment (Figure 1d,e), although HFD feeding or semaglutide treatment generated no appreciable effect on plasma adiponectin level in our current experimental settings (Figure 1f). Plasma FGF21 levels were comparable between LFD‐fed and HFD‐challenged mice, while its level was elevated in mice received 7‐day semaglutide treatment (Figure 1g). Fat weights (eWAT, iWAT, and BAT) and fat weight to body weight ratios were significantly elevated after HFD challenge (Figure 1h–m). Seven‐day semaglutide treatment reduced eWAT and iWAT weight moderately but generated no appreciable effect on reducing BAT level or BAT weight to body weight ratio (Figure 1h–m). We conclude that both glucose disposal and body weight lowering effects can be achieved by 7‐day semaglutide treatment.

### 3.2 Seven‐day semaglutide treatment restores HFD‐induced attenuation on ERK phosphorylation to hFGF21 treatment in hepatocytes

**FIGURE 2:**
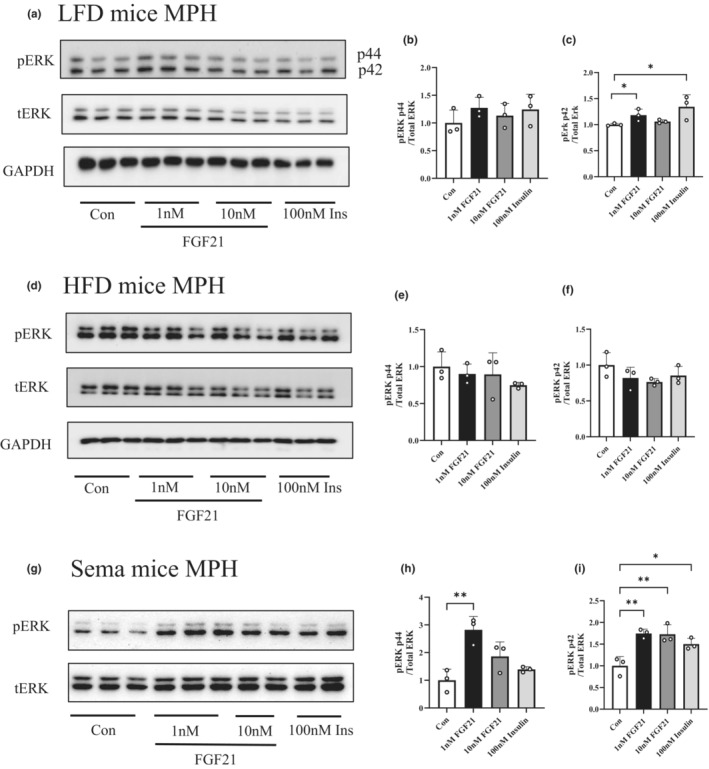
Seven‐day semaglutide treatment restores HFD‐induced attenuation on ERK phosphorylation to hFGF21 treatment in hepatocytes. (a) Western blotting show expression levels of indicated protein in MPH isolated from LFD‐fed mice after 1 h hFGF21 treatment with indicated dose. (b, c) Densitometric analyses for pERK (p44, Thr202) and pERK (p42, Tyr204) with indicated treatment. (d) Western blotting show expression levels of indicated protein in MPH isolated from HFD‐fed mice after 1 h hFGF21 treatment with indicated dose. (e, f) Densitometric analyses for pERK (p44, Thr202) and pERK (p42, Tyr204) with indicated treatment. (g) Western blotting show expression levels of indicated protein in MPH isolated from semaglutide‐treated mice after 1 h hFGF21 treatment with indicated dose. (h, i) Densitometric analyses for pERK (p44, Thr202) and pERK (p42, Tyr204) with indicated treatment. Data are shown as the mean ± SD. *p < 0.05, **p < 0.01, ***p < 0.001.

We then isolated primary hepatocytes from mice fed with either LFD, HFD, or HFD with 7‐day semaglutide treatment and tested their response to hFGF21 treatment. Figure 2a–c show results of Western blotting in the determination of effect of hFGF21 treatment on ERK phosphorylation in MPH isolated from LFD‐fed mice. One‐hour hFGF21 (either 1 or 10 nM) treatment generated no appreciable effect on AKT Ser473 (Supporting Figure S2A) or ERK p44 (Thr202) phosphorylation (Figure 2b), while AKT Ser473 phosphorylation can be effectively stimulated by 100 nM insulin treatment (Supporting Figure S2A). One nM FGF21 treatment, however, moderately stimulated ERK p42 (Tyr204) phosphorylation (Figure 2c).

The same experiments were then applied to MPH isolated from mice fed with HFD for 14 weeks (Figure 2d–f) and for HFD‐challenged mice with 7‐day semaglutide treatment (Figure 2g–i). The stimulatory effect of hFGF21 treatment on ERK p42 Tyr204 phosphorylation was absent in MPH of HFD‐challenged mice (Figure 2e,f); while semaglutide treatment restored HFD‐induced impairment on ERK phosphorylation. Specifically, hFGF21 (1 and 10 nM) treatment stimulated both ERK p44 (Thr202) and p42 (Tyr204) phosphorylation (Figure 2h,i).

### 3.3 Seven‐day semaglutide treatment restores the stimulatory effects of hFGF21 on its downstream target gene expressions in MPH

**FIGURE 3:**
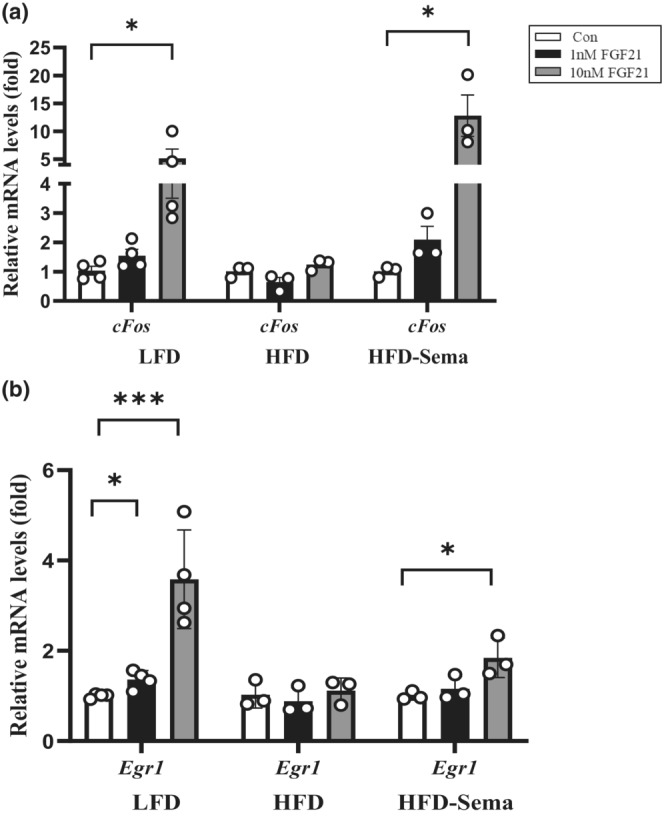
Seven‐day semaglutide treatment restores the stimulatory effects of hFGF21 on its downstream target gene expressions in MPH. (a) qRT‐PCR show effect of indicated dose of hFGF21 treatment (4 h) on expression of *cFos* in MPH isolated from LFD‐fed, HFD‐fed, and semaglutide‐treated mice. (b) qRT‐PCR show effect of indicated dose of hFGF21 treatment (4 h) on expression of *Egr1* in MPH isolated from LFD‐fed, HFD‐fed, and semaglutide‐treated mice. Data are shown as the mean ± SD. *p < 0.05, **p < 0.01, ***p < 0.001.

We then assessed FGF21 downstream target gene expressions including *cFos* and *Egr1* in MPH of LFD, HFD and HFD with seven‐day semaglutide treatment. Isolated MPH was treated with 1 nM or 10 nM of hFGF21 for 4 h for gene expression analysis. As shown, 10 nM (but not 1 nM) hFGF21 treatment stimulated expression of *cFos*, while such stimulatory effect was lost in MPH of HFD‐fed mice. Seven‐day semaglutide treatment reversed HFD‐induced attenuation on *cFos* expression (Figure 3a). *Egr1* is another defined downstream target gene for FGF21. Its expression was elevated by both 1 and 10 nM of hFGF21 treatment in MPH of LFD‐fed mice. In MPH of HFD‐fed mice, no appreciable effects were observed on *Egr1* stimulation either by 1 nM or 10 nM hFGF21 treatment, while such impairment was partially restored by 7‐day semaglutide treatment. Specifically, 10 nM of hFGF21 treatment significantly elevated *Egr1* mRNA level (Figure 3b). Together, the above observations collectively suggest that short‐term semaglutide treatment restores HFD‐induced FGF21 signaling impairment in mouse hepatocytes.

### 3.4 Seven‐day semaglutide treatment stimulates hepatic FGF21 expression and expression of *Fgfr1* and *Klb*

**FIGURE 4:**
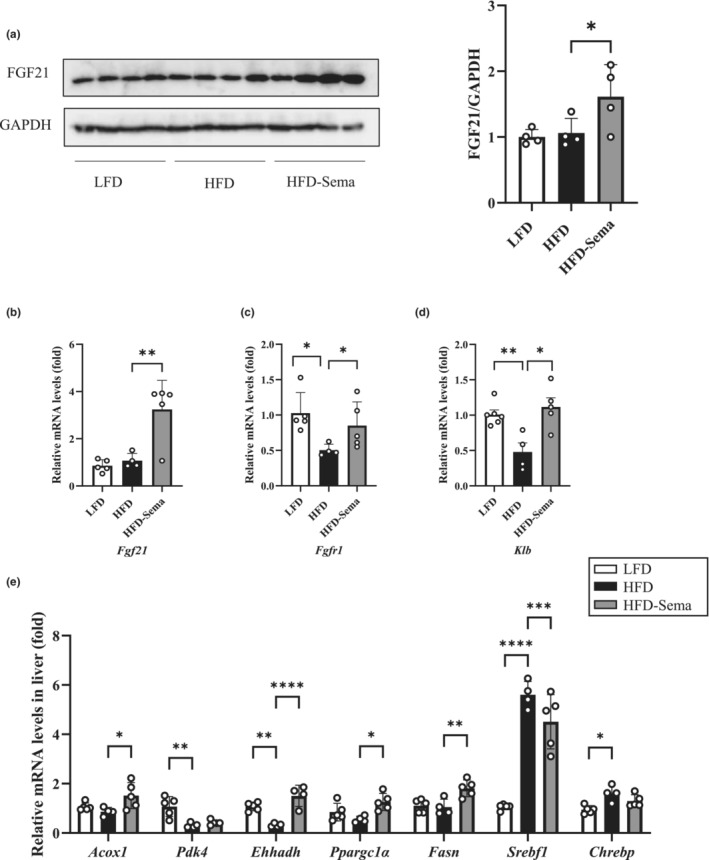
Seven‐day semaglutide treatment improves FGF21 sensitivity and attenuates the effect of HFD feeding on hepatic gene expression. (a) Western blotting show expression levels of FGF21 in the liver of three indicated groups. (b–d) qRT‐PCR shows the comparison of expression levels on *Fgf21* (b) and genes that encode its receptor, *Fgfr1* (c) and co‐receptor *Klb* (d) in the liver of three indicated groups. (e) qRT‐PCR shows the comparison of expression levels of lipogenic and fatty acid oxidation genes in the liver of three indicated groups. Data are shown as the mean ± SD. *p < 0.05, **p < 0.01, ***p < 0.001, ****p < 0.0001.

The liver tissues from the three groups of mice were then isolated for assessing FGF21 expression at both protein and mRNA levels. In the current study, HFD feeding did not significantly increase hepatic FGF21 levels, while daily semaglutide treatment for 7 days generated a significant stimulatory effect on FGF21 level, assessed by Western blotting (Figure 4a). Figure 4b shows that semaglutide treatment increased liver *Fgf21* mRNA level. Figure 4c,d show that HFD challenge‐induced attenuation on expression of Fgfr1 and Klb was reversed by 7‐day semaglutide treatment. T.

### 3.5 Seven‐day semaglutide treatment attenuates the effect of HFD feeding on hepatic gene expression

We have demonstrated previously that daily liraglutide treatment for 3 weeks can ameliorate HFD‐induced alterations on expression of genes that are involved in lipid homeostasis and fatty acid β oxidation in the liver (Liu et al., 2021). Here, we aimed to test whether short‐term semaglutide treatment can also process the similar effects. Expression of hepatic genes that are involved in lipogenesis and fatty acid β oxidation was assessed in the three groups of mice. Figure 4e shows that HFD feeding significantly reduced expression of genes that encode hepatic pyruvate dehydrogenase kinase 4 (*Pdk4*), and enoyl‐CoA hydratase and 3‐hydroxyacyl CoA dehydrogenase (*Ehhadh*). Furthermore, there was a trend of decrease on expression of the gene that encodes peroxisomal acyl‐CoA oxidase 1 (*Acox1*) after HFD challenge. Seven‐day semaglutide treatment increased expression levels of *Acox1* and *Ehhadh*, but not *Pdk4*. Semaglutide treatment also significantly increased the expression of *Ppargc1a*, which encodes the FGF21 downstream target peroxisome proliferator‐activated receptor γ coactivator 1 α, a key player in facilitating fatty acid β oxidation. HFD feeding also increased expression of the two genes that encode transcription factors of lipogenesis, namely sterol regulatory element‐binding transcription factor 1 (*Srebf1*) and carbohydrate response element‐binding protein (*Chrebp*). Seven‐day semaglutide treatment repressed their expression, although the repression on expression *Chrebp* is relatively moderate. HFD feeding generated no appreciable effect on expression of *Fasn*, which encodes fatty acid synthase. Seven‐day semaglutide treatment stimulated *Fasn* expression level (Figure 4e).

### 3.6 Seven‐day semaglutide treatment attenuates HFD‐induced alterations on a battery of adipose‐specific genes

**FIGURE 5:**
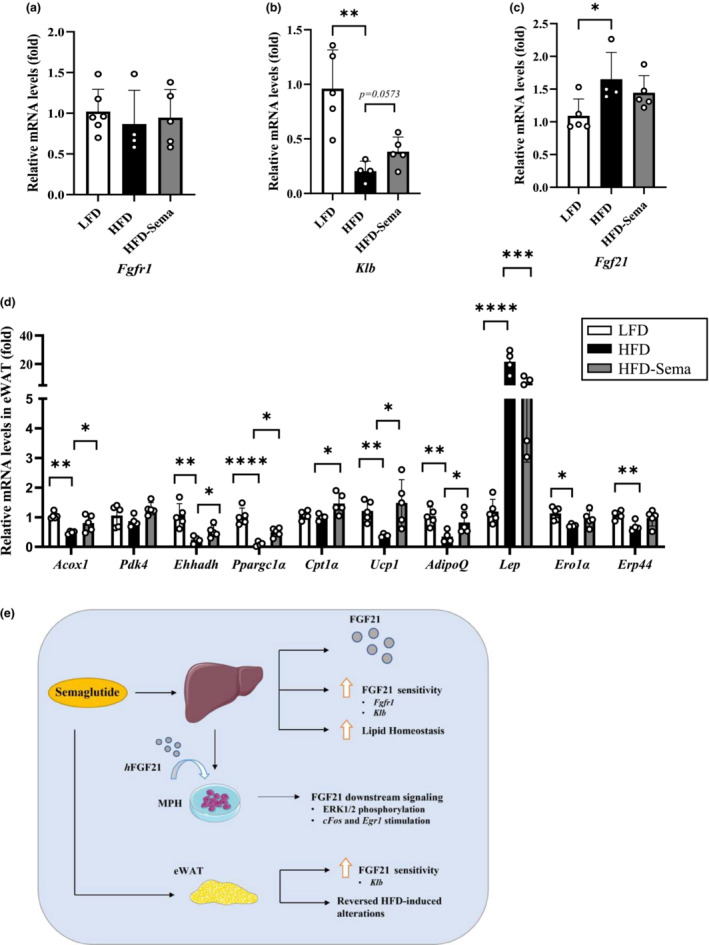
Seven‐day semaglutide treatment recovers HFD‐induced alteration in a battery of adipose‐specific genes. (a–c) qRT‐PCR shows the comparison of expression levels on *Fgfr1* (a), *Klb* (b) and *Fgf21* (c) in the eWAT of three indicated groups. (d) qRT‐PCR shows the comparison of expression levels of a battery of adipose tissue‐specific genes in the eWAT of three indicated groups. (e) The diagram shows the observed effects of short term semaglutide treatment on mice fed with HFD. In MPH, seven‐day semaglutide treatment restores the response to hFGF21 treatment. In the liver and eWAT, the treatment improves FGF21 sensitivity and restores HFD‐induced attenuation on genes that involved in maintaining lipid homeostasis and other adipose tissue‐specific genes. Data are shown as the mean ± SD. *p < 0.05, **p < 0.01, ***p < 0.001, ****p < 0.0001.

Finally, we assessed the effect of short‐term semaglutide treatment on epidydimal white adipose tissue (eWAT). In eWAT, semaglutide treatment also showed improvement in HFD‐induced FGF21 resistance by partially restoring *Klb* expression level. However, such elevation did not reach statistical significance. *Fgfr1* levels remained unaffected by HFD feeding or semaglutide treatment (Figure 5a,b). HFD feeding increased *Fgf21* expression level in eWAT while one‐week semaglutide treatment did not cause a further increase (Figure 5c). Figure 5d shows that HFD challenge significantly repressed *Acox1*, *Ehhadh*, and *Ppargc1a*, but not *Pdk4* expression levels. Seven‐day semaglutide treatment partially restored HFD‐induced alterations on these three genes. Furthermore, adipose tissue‐specific genes including those that encode leptin (*Lep*), adiponectin (*AdipoQ*), and UCP1 (*Ucp1*), were significantly altered by HFD challenge. HFD challenge increased *Lep* level by nearly 26‐fold, and such elevation was partially attenuated by one‐week semaglutide treatment (reduced to ~6.5 folds). Such changes agreed with plasma leptin level changes presented in Figure 1e. *AdipoQ* level in eWAT, however, was significantly reduced after HFD challenge, and the reduction was partially restored by 7‐day semaglutide treatment. Such changes disagreed with unchanged plasma adiponectin hormone levels after HFD challenge or semaglutide treatment (Figure 1f). Due to the inconsistency between plasma adiponectin hormone levels and *AdipoQ* levels in eWAT, we then assessed two chaperone genes known as *Ero1a* and *Erp44* in eWAT. These two genes encode for endoplasmic reticulum oxidoreductase 1 alpha and endoplasmic reticulum protein 44, respectively; involved in mediating oligomerization of the active high molecule weight form of adiponectin. Both *Ero1α* and *Erp44* levels were significantly repressed by the HFD challenge. Following one‐week semaglutide treatment, their expression levels were comparable with that in LFD‐fed mice (Figure 5d).

## 4 DISCUSSION

Shortly after GLP‐1 was recognized as an incretin, extra‐pancreatic functions of this gut hormone including that in the liver and adipose tissues have been broadly studied and recognized (Jin & Weng, 2016). Functions of GLP‐1 and GLP‐1RAs in the liver as well as in adipose tissues have provided plausible explanations for their effectiveness in treating T2D subjects with severe insulin resistance as well as their profound effect on lipid homeostasis (Hein et al., 2013; Jin & Weng, 2016; Taher et al., 2014). For their hepatic functions, we have paid close attention to the hepatic hormone FGF21, as this hormone is actively involved in both glucose and lipid homeostasis (Badakhshi & Jin, 2021; Liang et al., 2014). For exploring our mechanistic understanding on hepatic functions of GLP‐1 and GLP‐1RAs, we and others have revealed that in various rodent models, GLP‐1RAs including liraglutide and exenatide can stimulate hepatic FGF21 expression (Lee et al., 2014; Liu et al., 2019, 2021; Liu & Gao, 2019; Nonogaki et al., 2014; Yang et al., 2012). Here we show that short‐term semaglutide treatment can also stimulate hepatic FGF21 expression in HFD‐challenged C57BL/6J mice. More importantly, we expanded our investigation into FGF21 sensitivity in both the liver and eWAT, involving the restoration of HFD challenge‐induced repression on *Fgfr1* or *Klb*. We have also expanded our investigation on FGF21 signaling sensitization in response to GLP‐1RA treatment utilizing in vitro hFGF21 treatment on MPH.

In the diabetic *KKAy* mouse model, liraglutide treatment was shown to suppress both obesity and hyperglycemia, associated with increased hepatic FGF21 production (Nonogaki et al., 2014). In HFD‐diet challenged *ApoE* ^*−/−*^ mouse model, liraglutide treatment was also shown to stimulate hepatic FGF21 production, associated with improved insulin sensitivity (Yang et al., 2012). Another investigation demonstrated that daily exenatide treatment for 10 weeks increased expression of hepatic FGF21 and genes that encode its receptor and co‐receptor in HFD‐challenged mice (Lee et al., 2014). Furthermore, this study showed that in the human Hepa1‐6 cell line, in vitro exenatide treatment can directly increase FGF21 expression and mechanistically, the regulation is mediated by activating silent mating type information regulation 2 homolog (SIRT1) (Lee et al., 2014). We, however, cannot reproduce such in vitro effect with direct liraglutide treatment in MPH (Liu et al., 2021). Exenatide stimulated FGF21 production was also observed in obese *db/db* mice as well as in *Pax6* (*m*/+) mice (Liu et al., 2019). In a recent study, we were unable to observe the in vitro stimulatory effect of liraglutide treatment on FGF21 production in MPH, which agrees with the lack of GLP‐1R detection in mouse liver, utilizing RNA‐seq and other tools (Liu et al., 2021). The lack of GLP‐1R in mouse liver was also reported by other investigations (Baggio et al., 2018; Panjwani et al., 2013). Furthermore, we demonstrated that liraglutide cannot stimulate hepatic FGF21 in GLP‐1R KO mice and that in liver specific FGF21 KO mice, liraglutide treatment showed virtually no metabolic beneficial effects, especially on lipid homeostasis (Liu et al., 2021). Thus, liraglutide utilizes GLP‐1R expressed elsewhere to stimulate hepatic FGF21 expression, and such stimulation is patho‐physiologically important. To identify such extra‐hepatic organ would require the generation of tissue specific GLP‐1R KO mouse models during adulthood.

FGF21 exerts pleiotropic metabolic beneficial effects including the increase of insulin sensitivity, the facilitation of energy expenditure, as well as the decrease in body weight and glucose uptake by adipocytes (Badakhshi & Jin, 2021). The term “FGF21 resistance” was initially coined to describe the phenomena in obese mice, showing decreased expression of FGF21 receptor complex in eWAT, which was associated with increased plasma FGF21 level, blunted ERK phosphorylation and attenuated reduction in plasma glucose level in response to exogenous FGF21 administration (Badakhshi & Jin, 2021; Fisher et al., 2010). The paradoxical relationship between plasma FGF21 level and obesity was also interpreted as the development of FGF21 resistance in obese subjects (Fisher et al., 2010). We have reported that HFD feeding can induce hepatic FGF21 resistance and such effect can be attenuated by concomitant dietary intervention with the polyphenol curcumin (Zeng et al., 2017). In that study, we have established the method for assessing hFGF21 treatment on MPH on ERK phosphorylation as well as FGF21 downstream target gene expression. Briefly, MPH from mice fed with HFD for 12 weeks developed FGF21 resistant, which can be attenuated by 12‐week concomitant curcumin intervention (Zeng et al., 2017). In our most recent studies, we expanded the investigation on hepatic FGF21 expression in response to GLP‐1RA treatment, including liraglutide and the long‐term effective GLP‐1RA semaglutide (Liu et al., 2021, 2022). Surprisingly, 4‐week liraglutide treatment but not semaglutide treatment showed the stimulatory effect on hepatic FGF21 expression.

In our view, potential explanations for the lack of stimulation on hepatic FGF21 by semaglutide treatment are as follows. Firstly, we suggested that GLP‐1RAs activate hepatic FGF21 expression via an extra‐hepatic organ that does express GLP‐1R (Liu et al., 2021). The two drugs may target such target organ with different efficacy. For example, if the target organ is the brain, the two drugs might penetrate blood brain barrier differently. Secondly, GLP‐1RAs may stimulate both hepatic FGF21 expression and FGF21 sensitivity, and the long‐term effective semaglutide may exert such functions more effectively, leading to the triggering of a negative feedback on hepatic FGF21 expression. In other words, we may have missed the “activation window” after semaglutide treatment for 4 weeks. We assessed the second possibility in current study by testing the effect of short‐term semaglutide treatment. Partially for this reason, we have increased the dosage of semaglutide to 600 μg/kg body weight. As shown, 7‐day semaglutide treatment significantly reduced body weight and repressed food intake in mice with HFD challenge. In addition, 7‐day semaglutide treatment increased serum FGF21 level, hepatic FGF21 hormone level as well as hepatic *Fgf21* mRNA level and that MPH isolated from HFD‐fed mice received 7‐day semaglutide treatment showed restored sensitivity to in vitro hFGF21 treatment on ERK phosphorylation and FGF21 target gene expression, and that in those mice both liver and eWAT showed partially restored expression of *Klb*. Like 4‐week liraglutide treatment (Liu et al., 2021), 7‐day semaglutide treatment also restored expression of *Fgfr1* in the liver, which was attenuated by HFD challenge. In eWAT, either HFD challenge or 7‐day semaglutide treatment generated no appreciable effect on *Fgfr1* expression level, which agrees with an early study conducted by Yang and colleagues (Yang et al., 2012). GLP‐1R activation has been shown to improve glycemic control and promote satiety, leading to reduced caloric intake and body weight (Baggio & Drucker, 2014; Drucker, 2022). Due to high dosage utilized in current study, seven‐day semaglutide treatment may induced a “fasting‐like” state in mice with HFD‐challenged. Since FGF21 is a fasting hormone, the increased level can be a results of reduced food intake (Fazeli et al., 2015; Zhang et al., 2012). Hence, whether semaglutide treatment‐induced stimulation on FGF21 level is secondary to its anorexigenic effect require further investigation.

We have reported the stimulatory effect of 3‐week liraglutide treatment in high fat and high fructose diet challenged mice on expression of *Ppargc1a*, *Acox1*, *Pdk4*, and *Ehhadh*, and the repressive effect of its treatment on *Screbf1* in the liver (Liu et al., 2021). Those regulatory effects can be reproduced in HFD‐challenged mice with 7‐day semaglutide treatment. Among them the product of *Ppargc1a* directly mediates function of FGF21 in lipid homeostasis, while others are involved in lipogenesis and fatty acid β oxidation. In eWAT, we show here that *AdipoQ* level was repressed by HFD challenge, and the repression was attenuated by 7‐day semaglutide treatment. In mouse plasma, however, adiponectin hormone level was not significantly affected by 14‐week HFD feeding or 7‐day semaglutide treatment. The difference could be due to post‐translational modifications of the adiponectin hormone. We hence assessed the effect of HFD challenge and 7‐day semaglutide treatment on genes known as *Ero1α* and *Erp44*, which encode for the two chaperones that are engaged in adiponectin oligomerization. We show here that HFD feeding reduced expression of these two chaperone genes, whereas semaglutide treatment was able to restore their expression levels. Low plasma adiponectin level is linked to insulin resistance and increased T2D incidence. It has been reported that FGF21 stimulates adiponectin secretion in adipocytes (Lin et al., 2013). Thus, in HFD‐challenged mice, semaglutide mediated stimulation on *AdipoQ* expression and restoration of *Ero1α* and *Erp44* expression may still be physiologically important. Further investigations are needed to assess plasma total adiponectin level, adiponectin oligomerization, and active adiponectin level, in obese mice treated with GLP‐1RAs during different time intervals.

To clarify the effect of semaglutide on hepatic FGF21 level, we designed the short‐term treatment of 7‐day, with a high dosage (600 μg/kg body weight). Such high dosage treatment did not generate obvious abnormalities on mouse health, including the development of hypoglycemia. Clinically, high dose weekly semaglutide treatment has shown to exert better outcome in body weight lowering and glucose disposal (Bradley et al., 2022; Frías et al., 2021). We have learned for decades that early intensive insulin therapy in patients with newly diagnosed T2D can bring favorable outcomes (Weng et al., 2008), while in T2D subjects with severe insulin resistance, high dose insulin treatment can be well‐tolerated and effective on improving glucose disposal (Kampmann et al., 2011). It is worth testing whether early intensive GLP‐1RA therapy can bring a better therapeutic effect in early diagnosed T2D patients.

Numerous studies have reported the metabolic regulatory role of Sirt1 in lipid homeostasis (Hou et al., 2008; Majeed et al., 2021; Simmons et al., 2015). Specifically, Sirt1 was determined to play a beneficial role in protecting HFD‐induced or alcohol consumption‐induced hepatic steatosis, yet the mechanism underlying its metabolic functions is not fully understood (Pfluger et al., 2008). Weng et al. has showed that Sirt1 mediates the effects of GLP‐1RA exenatide on attenuating hepatic steatosis (Xu et al., 2014). DPP4i, vildagliptin has been revealed to induce FGF21 via Sirt1 signaling (Furukawa et al., 2021). Thus, it is worthwhile to examine whether Sirt1 plays a role in improving FGF21 sensitivity after GLP‐1RA treatments. Here, we choose male mice as our animal model since female mice are known to be resistant against HFD feeding. In a recent study, we have determined that hepatic FGF21 expression is regulated by estrogen‐Wnt signaling cascade (Badakhshi et al., 2021). Hence, a technical breakthrough is required in the field to better understand the involvement of female hormone in current study.

Figure 5e summarizes our results in current study. In obese mice, 7‐day high dose semaglutide treatment increased hepatic FGF21 level, associated with attenuated repression on expression of *Fgfr1* and *Klb* in the liver. In addition, alterations on a battery of genes that are implicated in mediating functions of FGF21 in the liver induced by HFD challenge were attenuated by 7‐day semaglutide treatment. In MPH, HFD‐induced impairments on FGF21 signaling were also restored by 7‐day semaglutide treatment. In eWAT, 7‐day semaglutide treatment partially restored the repressive effect of HFD on *Klb* expression and effectively restored the repression of HFD on *Acox1*, *Ehhadh*, and *Ppargc1α*, downstream effectors of FGF21. We hence confirmed that the GLP‐1RA semaglutide can up‐regulate hepatic FGF21 production and restore FGF21 sensitivity that is impaired by HFD challenge. Mechanisms underlying effect of short‐term semaglutide treatment on eWAT hormone gene expression and the restoration of expression of the two chaperone genes impaired by HFD challenge are also worth to be further examined.

## AUTHOR CONTRIBUTIONS

JNF, WS, and TJ conceived and designed research. JNF and WS performed experiments. JNF analyzed data. JNF, WS, and TJ interpreted results of experiments. JNF and WS prepared figures and tables. JNF drafted the manuscript. TJ edited and revised the manuscript.

## FUNDING INFORMATION

This study is supported by the Canadian Institutes of Health Research (PJT159735 to T.J.).

## CONFLICT OF INTEREST STATEMENT

The authors declare no competing interests.

## DISCLAIMERS

JNF is a Ph.D. student supported by the Banting & Best Diabetes Centre (BBDC)‐Novo Nordisk Studentship, and Canada Graduate Scholarships—Master's program (CGS M).

## Supporting information


Supplemental Figure 1.
