# Supplementary material for: Short‐term semaglutide treatment improves FGF21 responsiveness in primary hepatocytes isolated from high fat diet challenged mice
Source: Physiol Rep. 2023 Mar 10;11(5):e15620. doi: 10.14814/phy2.15620 (PMC10006666; doi:10.14814/phy2.15620)

**Supplemental Figure Legends**

**Supplemental Figure 1.** Seven-day semaglutide treatment reduced body weight and body weight gain. A) Body weight changes during 14-week of experiment period in the three indicated groups. B) Body weight gain from week 1 to 14 in the three indicated groups. C) Food intake during 14-week experimental period in the three indicated groups. D-E) Liver weight (D) and Liver weight to body weight ratio (E) in the three indicated groups. Data are shown as the mean ± SD. *P < 0.05. **P < 0.01, ***P < 0.001.

**
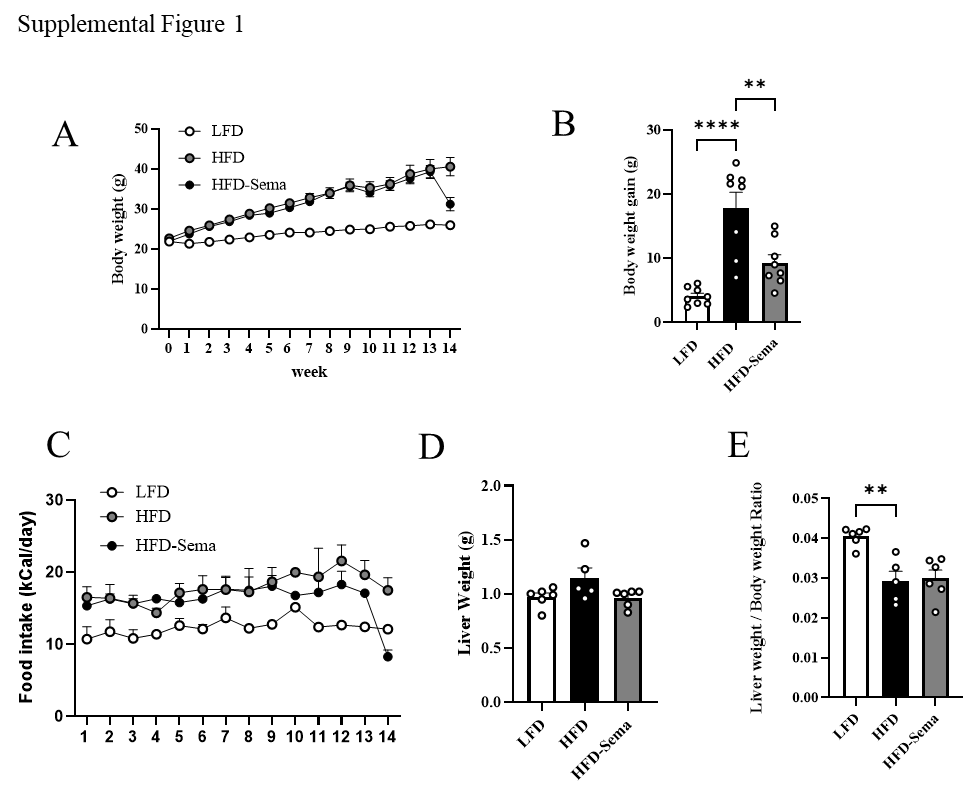
**

**Supplemental Figure 2.** 14-week HFD shows reduced AKT phosphorylation level of MPH to insulin treatment. A) Western blotting show expression levels of pAKT in MPH isolated from LFD-fed mice after 1h of indicated treatment with indicated dosage**.** B) Densitometric analyses for pAKT with indicated treatment. C) Western blotting show expression levels of pAKT in MPH isolated from HFD-fed mice after 1h of indicated treatment with indicated dosage**.** D) Densitometric analyses for pAKT with indicated treatment. Data are shown as the mean ± SD. *P < 0.05. **P < 0.01, ***P < 0.001.


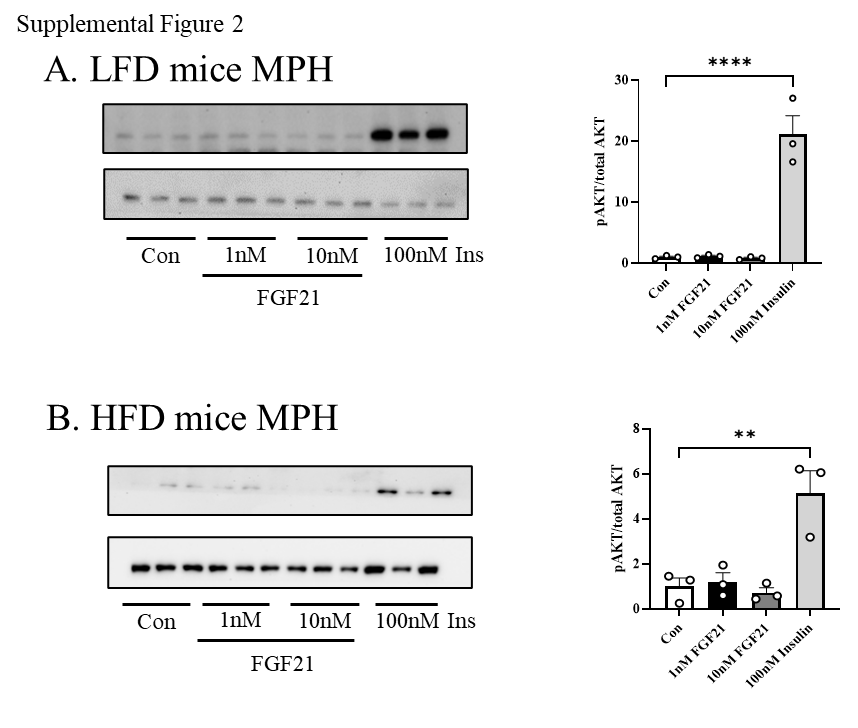

Supplement: Supplementary file 1 — Supplemental Figure 1. [file PHY2-11-e15620-s001.docx]
